# Molecular Detection and Isolation of *Bartonella* Species in Bats and Their Ectoparasites Along the China–Myanmar Border

**DOI:** 10.1155/tbed/5517852

**Published:** 2025-08-25

**Authors:** Chenjie He, Yuhong Chen, Yin Yang, Peiyu Han, Wei Kong, Song Wu, Yun Long, Junying Zhao, Ze Yang, Bo Wang, Yunzhi Zhang

**Affiliations:** ^1^Institute of Preventive Medicine, School of Public Health, Dali University, Dali, Yunnan, China; ^2^Key Laboratory for Cross-Border Control and Quarantine of Zoonoses in Universities of Yunnan Province, Dali, Yunnan, China; ^3^Department of Medical, The Second People's Hospital of Dali Prefecture, Dali, Yunnan, China; ^4^Programme in Emerging Infectious Diseases, Duke-NUS Medical School, Singapore, Singapore

**Keywords:** *Bartonella*, *Bartonella* isolation, bats, *Candidatus Bartonella dianxisis*, China–Myanmar border, ectoparasites, genetic diversity, tissue tropism

## Abstract

*Bartonella* are parasitic pathogens that infect many mammals, including humans, and cause significant diseases. This study investigates the presence, genetic diversity, and tissue tropism of *Bartonella* in bats and their ectoparasites along the China–Myanmar border. Bats and ectoparasites were collected from Yingjiang, Ruili, and Gengma Counties. Nested PCR (nPCR) and quantitative real-time PCR (qPCR) were used to detect and quantify *Bartonella* in bat tissues. *Bartonella* was isolated using brain–heart infusion broth and tryptone soy agar medium containing 5% sheep blood (TSA containing 5% sheep blood), and DNA sequences were analyzed with Clustal W and MEGA X. In total, 601 bats from 11 species (four families and seven genera) and 32 ectoparasites (two orders, three families, and four genera) were collected. The qPCR results revealed *Bartonella* detection rates of 22.96% (138/601) in bats and 62.50% (5/8) in ectoparasites. Using nPCR to detect the *Bartonella gltA* and *rpoB* genes in bats, ectoparasites, and strains isolated from bat blood samples, yielding 58 and 10 strains, respectively. When comparing bats, ectoparasites, and isolated strains to other *Bartonella* in GenBank, the *gltA* gene was 74.21%–100.00% at the nucleotide level of similarity and 75.70%–100.00% at the amino acid level. In comparison, the *rpoB* gene was 79.58%–100.00% at the nucleotide level of similarity and 89.71%–100.00% at the amino acid level. By phylogenetic analysis except for *Bartonella* sp. and uncultured *Bartonella* sp., we found a clade that was less than 96.0% at the nucleotide level of similarity in the *gltA* gene and less than 95.4% at the nucleotide level of similarity in the *rpoB* gene. Based on the threshold values for the delineation of new species of *Bartonella*, we believe that a new species of *Bartonella* prevalent in bats was discovered in this study, which we named “*Candidatus Bartonella dianxisis*”. Otherwise, the average copy number of *Bartonella* in bat tissues (blood, spleen, heart, brain, kidney, lung, liver, and rectum) ranged from 1.15 × 10^4^ to 6.87 × 10^4^ copies/μL, with the highest levels observed in blood and spleen. Our findings highlight the genetic diversity of *Bartonella* in bats and ectoparasites along the China–Myanmar border and underscore potential public health risks associated with these pathogens.

## 1. Introduction

*Bartonella* is a genus of gram-negative bacteria that primarily infects endothelial cells and erythrocytes. It belongs to the phylum Bacteria, class Alphaproteobacteria, order Hyphomicrobiales, and family Bartonellaceae. Bartonellosis is an emerging and re-emerging infectious disease with a broad spectrum of clinical manifestations. Symptoms can range from mild ones like fever, headache, and malaise to more severe effects such as hallucinations. The bacteria are primarily transmitted among mammals by arthropods and are responsible for several human illnesses, including cat-scratch disease (CSD), trench fever, endocarditis, and lymphadenitis [1].

There are currently 39 known species and three subspecies of *Bartonella* worldwide [2] (https://www.bacterio.net/genus/bartonella, assessed on October 24, 2024), with numerous unnamed and unidentified species still to be described. Seventeen *Bartonella* species have been linked to human illness [1], including *B. henselae* [3], *B. tribocorum* [4], *B. quintana* [5], *B. grahamii* [6], *B. rochalimae* [7], and *B. elizabethae* [8]. The primary natural hosts of *Bartonella* species are mammals from several taxonomic orders, including Carnivora (e.g., cats, dogs, and foxes) [9], Perissodactyla (e.g., horses), Artiodactyla (e.g., cows, sheep) [10, 11], Primates (e.g., rhesus monkeys) [12], Rodentia [13], and Chiroptera [14]. The order Chiroptera, which includes bats, is the second largest group in the class Mammalia, after Rodentia. With 1462 species classified into 21 families and 264 genera, bats represent over 25% of all mammal species (https://www.mammalwatching.com/resources/global-mammal-checklist/, assessed on October 24, 2024). As the only group of mammals capable of flight, bats are uniquely suited to carry and transmit a wide variety of pathogens, making them important vectors for *Bartonella* and other zoonotic diseases [15].

Bats have a wide geographical distribution worldwide and are often associated with emerging pathogens due to their unique immune mechanisms [16]. While much research has focused on bats as reservoirs of viral pathogens, less attention has been given to bacterial pathogens such as *Bartonella* [17]. More than 100 species of bats worldwide have been identified as hosts of *Bartonella* [18]. It is believed that bats were the original hosts of *Bartonella* and may have played a vital role in the early geographic spread of the genus [19]. A case report described a *Bartonella* infection of bat origin that caused endocarditis in a male patient from the USA [20, 21]. Studies of bat-derived *Bartonella* in Georgia revealed a high genetic to strains isolated from human samples in Poland and dog samples in Thailand [17]. These findings highlight that *Bartonella* is widely distributed in bats, likely due to factors such as host specialty and coevolution. The bacteria have been isolated from bats in various Countries, For example, Bai et al. [16] isolated and characterized *Bartonella rousetti* from *Rousettus aegyptiacusa* in Nigeria; Nabeshima et al. [22] isolated and studied the *Bartonella* in *Miniopterus fuliginosus* from Japan and found that Japan contains two endemic *Bartonella* of *Miniopterus*; In addition, a study of *Bartonella* in bats from Thailand isolated bacteria belonging to the *Bartonella* species from five bat species (including *Chaerephon plicatus*, *Hipposideros armiger*, *Hipposideros fulvus*, *Hipposideros larvatus*, and *Taphozous melanopogon*) [23]. Bat ectoparasites, such as bat flies, ticks, fleas, and mites, are believed to play a significant role in the transmission of *Bartonella* among bats [17]. Therefore, studying the interactions between *Bartonella*, bats, and their ectoparasites is essential for understanding the ecology and transmission of this pathogen.

The *ssrA* gene is a small RNA molecule found in bacteria that plays a key role in protein modification [24]. The *ssrA* genes in *Bartonella* are valuable tools for the rapid identification and classification of these bacteria [25]. Since the 1980s, various molecular techniques, such as DNA–DNA hybridization, multilocus sequence analysis (MLSA), and single-gene assays, have been developed to classify *Bartonella* species [26]. Traditional PCR methods, however, have limitations, including high detection limits and low sensitivity and specificity. To overcome these challenges, this study employed quantitative real-time PCR (qPCR) to detect the *ssrA* gene, a single-copy prokaryotic-specific marker, allowing for improved sensitivity and specificity in *Bartonella* detection. Scola et al. [27] carried out a comparative analysis of seven different genes in *Bartonella* and showed that genes encoding RNA polymerase subunits (*rpoB*) and citrate synthase (*gltA*) genes for intraspecific differentiation proved to be the most efficient. Therefore, we also amplified and characterized the species-specific *gltA* and *rpoB* genes of *Bartonella* to understand the genetic evolution of *Bartonella* on bats and their ectoparasites in our region by nPCR. We investigated the presence of *Bartonella* in bats from three counties along the China–Myanmar border (Yingjiang, Ruili, and Gengma), analyzed the genetic diversity of the *Bartonella*, and quantitatively assessed their presence in bat blood, brain, heart, lung, liver, spleen, kidney, and intestinal tissues. Additionally, we isolated and characterized *Bartonella* strains.

## 2. Materials and Methods

### 2.1. Medical Ethics Approvals

This research was approved by the Medical Ethics Committee of Dali University under number 2021-PZ-177. All animals were treated according to the Guidelines of Regulations for the Administration of Laboratory Animals (Decree No. 2 of the State Science and Technology Commission of the People's Republic of China, 1988) and the Guidelines for Treating Animals Kindly from Ministry of Science and Technology of the People's Republic of China. All efforts were made to minimize discomfort to the animals.

### 2.2. Sample Collection

**Figure 1:**
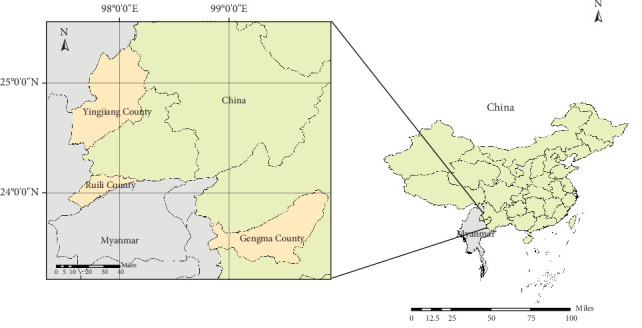
Map of sampling locations. The right panel shows the geographical map of China and Myanmar, while the left panel highlights the counties and cities situated along the China–Myanmar border region where sampling was conducted.

Bat samples were collected from Yingjiang County, Ruili City, and Gengma County, located along the China and Myanmar border, between July 2022 and August 2023. (Figure 1) Local villagers captured and culled bats in stages due to concerns about the potential damage caused by bat colonies to crops and fruit trees. After a certain number of bats were collected at various locations, they were stored in a refrigerator at 4°C before being transported back to the local CDC laboratory, where sample preparation was completed within 5 h of collection. During the dissection of executed bats in the autopsy laboratory, the harvested bats were first morphologically identified and numbered, and each bat was examined for the presence of ectoparasites, which were immediately extracted with forceps and stored according to the number in 2 mL cryogenic vials (DNase free) (CORNING, Shanghai, China), and the bats were dissected shortly after the harvest, and the blood clots in their thoracic cavities were collected using a pipette Pasteur (Servicebio, Wuhan, China) under aseptic conditions and stored in 1.5 mL microtube (DNase free; Servicebio, Wuhan, China). The bats' heart, lung, liver, spleen, kidney, rectum, and brain tissues were then collected in correspondingly numbered 2 mL cryogenic vials (DNase free), and all samples were stored in a removable −80°C refrigerator at our research center before being transported back to the testing center for analysis.

### 2.3. DNA Extraction of Bats

In the nucleic acid extraction laboratory, 1 g of each sample (blood, heart, liver, spleen, lung, kidney, rectum, and brain tissues) was clipped into small pieces using autoclaved surgical scissors. The samples were placed into GeneReady Animal PIII pulverizing tubes (Life Real, Guangzhou, China). Each tube was treated with 600 μL of phosphate buffer solution (PBS) and located into the GeneReady Ultimate Grinder (Life Real, Guangzhou, China) for mechanical grinding. Using a pipette, 300 μL of supernatant from the tissue suspension was transferred to the DNA extraction kit (TIANGEN, Beijing, China) and PBS was used as a blank control in the experimental manipulation. Nucleic acids were extracted using a fully automated nucleic acid extraction and purification system (BIOER, Hangzhou, China) according to the manufacturer's instructions. The extracted nucleic acid samples were aliquoted into two portions and stored at −80°C refrigerator for further analysis.

### 2.4. qPCR and Nested PCR (nPCR)

#### 2.4.1. qPCR Detection of *Bartonella* sp. in Bats

To detect *Bartonella* nucleic acids in bat tissues, a qPCR assay targeting the *ssrA* gene was performed, based on the work of Diaz et al. [25]. The details of the reaction system, cycling conditions, and oligonucleotides for qPCR can be found in Supporting Information 1: Table S1. A sample was considered positive for *Bartonella* if the circle threshold (Ct) was ≤38, based on a standard curve from plasmid standards. To prevent contamination and false positives, ddH_2_O was used as a blank control. Reaction preparation, sample pipetting, and amplification were conducted in separate areas.

#### 2.4.2. nPCR of *Bartonella* sp. in Bats

Samples that tested positive by qPCR were examined further, with the target *gltA* gene amplified using nPCR in accordance with the methods of Norman et al. [28] and Birtles and Raoult [29]. Semi-nested primers developed by Renesto et al. [30] and André et al. [31] were also used to amplify the target *rpoB* gene in positive samples. The final amplicon lengths were 357 bp and 603 bp, respectively. Specific descriptions of the oligonucleotides, cycling conditions, and reaction systems for nPCR are shown in Supporting Information 1: Table S1. During the experiment, ddH_2_O was used as a blank control, do not have positive control and the rest of the notes were similar to those for qPCR. The amplification products were separated on a 1.5% agarose gel (TransGen Biotech, Beijing, China), which was stained with the nucleic acid dye SuperRed (Biosharp, Beijing, China), and then visualized with a gel imaging system. Target bands were excised, purified with a purification kit (OMEGA Bio-Tek, Norcross, GA, USA), and sequenced by Sangon Biological Engineering Technology and Services Company Ltd. (Sangon Biotech) (Shanghai, China) Company.

#### 2.4.3. Molecular Identification of Bat Species

Bat species were initially identified morphologically in the field, followed by molecular confirmation in the laboratory using mitochondrial cytochrome b (*mt-Cytb*) amplification. Primers used were *Cytb*-F (5′-ATGATATGAAAAACCATCGTTG-3′) and *Cytb*-R (5′-TTTCCNTTTCTGGTTTACAAGAC-3′) [32–34]. The PCR reaction consisted of 25 µL containing 12.5 µL of 2 × Rapid Taq Master Mix, 1 µL each of *Cytb*-F and *Cytb*-R (10 µM), 9.5 µL of RNase-free ddH_2_O, and 1 µL of DNA template. The thermocycling program included predenaturation at 94°C for 3 min, followed by 40 cycles of denaturation at 94°C for 30 s, annealing at 51.9°C for 15 s, and extension at 72°C for 1 min and 20 s. A final extension at 72°C for 5 min completed the reaction. Amplicons (approximately equal to 1140 bp) [33], and the products were subjected to agarose gel electrophoresis and observed using a gel imaging system. Samples with correctly positioned electrophoretic bands were then selected and sent to Sangon Biotech for sequencing.

#### 2.4.4. DNA Extraction, Molecular Identification, and Detection *Bartonella* sp. of Ectoparasites in Bat

According to Kuang et al. [35], we first removed the samples from cryogenic vials at −80°C, then removed single stored bat ectoparasites from cryogenic tubes, rinsed twice using Minimum Essential Medium (MEM, Thermo Fisher, USA), discarded the rinsing solution, and placed the worms into grinding tubes, then added 300 µL of fresh MEM, and then ground using a grinder. Based on morphological identification and location information, the 32 individual worm grindings obtained were combined into eight reaction pools. Accordingly, 28 ectoparasites of *Nycteribiidae* were mixed into six pools, three ectoparasites of *Streblidae* were mixed into one pool and one tick in a separate pool (Table 1). A total volume of 300 μL of the pools mixing homogenate was pipetted into the DNA Extraction Kit (TIANGEN, Beijing, China). Nucleic acids were extracted using a fully automated nucleic acid extraction and purification system (BIOER, Hangzhou, China). The extracted nucleic acid samples were divided into triplicates and stored separately in a −80°C refrigerator for further analysis.

In molecular identification, mitochondrial cytochrome oxidase subunit I (*COI*) gene amplification was conducted using primers LCO1490 (5′-GGTCAACAAATCATAAAGATATTGG-3′) and HCO2198 (5′-TAAACTTCAGGGTGACCAAAAAATCA-3′) [36]. After performing nPCR on the grouped reaction pools, we obtained positive sample sequences for the *gltA* and *rpoB* genes for further analysis.

### 2.5. Sequence Comparison and Phylogenetic Analysis

Purified products were sequenced by Sangon Biotech using *gltA* and *rpoB* gene primers. The resulting sequences were assembled using the DNAstar Lasergene software package, manually edited, and trimmed to generate the final sequences. These sequences, derived from bats, arthropods, and pathogenic bacteria isolated in this study, were compared to *gltA* and *rpoB* gene sequences of *Bartonella* species available in the GenBank database using BLAST (http://blast.ncbi.nlm.nih.gov/Blast.cgi, assessed on October 24, 2024).

The *gltA* and *rpoB* gene reference sequences were downloaded from GenBank and a phylogenetic tree was constructed using the maximum likelihood method in MEGA X software [37]. First, the sequences obtained in this study were compared with the reference sequences in GenBank using Clustal W for the *gltA* gene. Then, the tree was constructed in MEGA X using the T92 + G model (Tamura 3-parameter + Gamma distribution) and *Brucella abortus* strains (CP081403.1 and CP081431.1) as the outgroup reference. The *rpoB* gene was then manipulated in the same way, and the most appropriate model (T92 + G) was chosen to build the tree and *B. abortus* strains (CP081443.1 and CP081377.1) as the outgroup reference. All sequences from this study were submitted to GenBank with the following accession numbers: *CytB*: PQ460893–PQ460958, PV667499–PV667524; *COI*: PQ461921–PQ461925, PV658813; *gltA*: PV695984–PV696039; *rpoB*: PV696040–PV696049.

### 2.6. Plasmid Construction and Standard Curves Plotting

A representative *Bartonella* strain was selected for cloning a fragment of its *ssrA* gene into the pEASY-T1 vector (TransGen Biotech, Beijing, China). The T-vector product was transformed into DH5-α *E. coli* cells, and positive single colonies were identified using a blue–white screening assay. Selected colonies were cultured in LB broth supplemented with 0.5% ampicillin (Soybean Casein Digest Agar; HuanKai Microbial, Guangzhou, China), and the target gene insertion was confirmed by sequence analysis. Plasmids containing the cloned fragments were extracted using the EasyPure Plasmid MiniPrep Kit (TransGen Biotech, Beijing, China), aliquoted, and stored at −20°C. The plasmid concentration was measured with a UV spectrophotometer (Life Real, Hangzhou, China) and converted into copy number using the formula are as follows:

  $$\begin{array}{c} \text{Copies}/µL=\frac{\text{Plasmind concentration }\left(\text{ng}/µL\right)\times 10^{-9}\times 6.02\times 10^{23}}{660\times \text{DNA length}}. \end{array}$$

A standard curve was established based on the plasmid copy number. A representative positive standard was serially diluted 10-fold and used as the template for a quantitative assay. Each concentration gradient was tested in triplicate, and the average Ct value was calculated. The standard curve was plotted with the logarithm of the plasmid copy number on the *x*-axis and the corresponding Ct values on the *y*-axis. The slope and correlation coefficient of the curve were determined to assess assay performance.

### 2.7. Tissue Tropism of *Bartonella* sp. in Bats

Quantification of *Bartonella* DNA in the blood and tissues of *Bartonella*-positive specimens was performed using the qPCR method described in this study to investigate *Bartonella* tissue tropism in bats.

### 2.8. *Bartonella* Cultivation and Identification

#### 2.8.1. Isolation of *Bartonella* sp. by Primary Culture of Blood

Our isolation method improved upon the method described by Liu et al. [38]. First, dilutions were configured by adding 5% (50 mg/mL) amphotericin B solution to brain–heart Infusion broth medium (BHI; HuanKai Microbial, Guangzhou, China) after autoclaving (121°C, 15 min), the BHI/AmpB ratio was set to 9:1. After being removed from the −80°C refrigerator, the blood was gently mixed and a 2 mL cryogenic vial was used to hold the inoculum solution. According to the numbering system, the blood homogenate and diluent were mixed in a 1:4 ratio to create an inoculum solution for spare use. Remove tryptone soy agar containing 5% sheep blood (TSA medium containing 5% sheep blood; Hopebio, Shandong, China), number it, and aspirate 100 μL of the inoculum solution. Inoculate the plate medium using the three-zone (or four-zone) inoculation ring method, then, once the inoculum had been absorbed by the medium, the plate was inverted and placed in a constant temperature incubator at 37°C with 5% CO_2_ for 30 days. Colony growth was observed daily, and the progression and size of colonies were recorded.

*Bartonella* colonies typically appear within 3–7 days but may require up to 1 month to develop fully [39]. Colonies are grayish-white or tan, small, round slightly transparent, and raised, with smooth or rough edges [40]. Primary cultures often contain stray bacteria or colonies with varying morphologies. Suspected *Bartonella* colonies were marked and isolated by streaking onto new TSA medium containing 5% sheep blood plates for subsequent incubation. This process was repeated for 1–6 generations to obtain pure colonies containing a single bacterial strain.

#### 2.8.2. Identification of Colonies

Although *Bartonella* is a gram-stain-negative bacterium, it does not easily adhere to gram stain dye. Thus, this study was conducted using Giemsa stain, which provides clear coloring and strong staining power for a better staining effect. The original solution (Solarbio Science & Technology [Beijing] Co., Ltd.) contains azure and eosin, which color acidophilic particles pink and basophilic particles blue–violet. Due to its unique characteristics, the Barton body combines with the azure in the Giemsa stain to reveal a blue–purple, rod- or ball-rod-shaped bacterial form under the microscope. So colonies were homogenized onto slides to form a monolayer, fixed, and stained using conventional Giemsa staining. Stained slides were under a light microscope using oil immersion at a magnification of 1000x (Axio Imager.A2, ZEISS, Germany Jena).

Using an inoculation needle, pick a single colony from the above purified colony plate and add it to a sterile microtube (DNase free) containing 100 μL of PBS. Mix well and in a water bath at 100°C for 10 min. Then centrifuge in an ultrahigh-speed centrifuge pre-cooled to 4°C at 12000 rpm for 10 min. Aspirate the supernatant into a new small microtube and store at −20°C. It can then be used as a template, and the *gltA* and *rpoB* genes can be amplified and identified.

### 2.9. Statistical Analysis

Statistical analyses were performed using *SPSS* 22.0 software. Chi-square and rank-sum tests were conducted to analyze the relationship between bat species, gender, body weight, and *Bartonella* infection rate. Multifactorial logistic regression analysis was used to identify risk factors associated with *Bartonella* detection rate (positive vs., negative). The model included all bat species with sample sizes of at least 10. Variables analyzed included bat species, gender, and body weight. Variables with *p*  < 0.1 were included in the model. Odds ratios (ORs) and 95% confidence intervals (CIs) were calculated to assess the strength of the associations. Tissue tropism results were compared across groups using analysis of variance (ANOVA) to evaluate differences in *Bartonella* distribution among tissues. Statistical significance was set at α = 0.05, with *p* < 0.05 considered significant.

## 3. Results

### 3.1. Characteristics of *Bartonella* in Bat Samples

A total of 601 bats from four families, seven genera, and 11 species were collected from Yingjiang, Ruili, and Gengma Counties along the China–Myanmar border (Figure 1). The most common species were *Miniopterus schreibersi* (34.61%, 208/601) and *H. armiger* (19.80%, 119/601). The overall detection rate of *Bartonella* detected by qPCR targeting the *ssrA* gene was 22.96% (138/601, 95% CI: 19.60%−26.30%). Species-specific prevalence rates were as follows: *M. schreibersi* (12.02%, 25/208), *H. armiger* (49.58%, 59/119), *Rousettus leschenaultia* (6.98%, 6/86), *Cynopterus sphinx* (27.03%, 20/74), *Rousettus amplexicaudatus* (19.51%, 8/41), *Miniopterus fuliginosus* (7.14%, 2/28), *H. larvatus* (60.00%, 12/20), *Eonycteris spelaea* (15.79%, 3/19), *Megaerops niphanae* (66.67%, 2/3), and *Rhinolophus blythi* (100.00%, 1/1).

**Figure 2:**
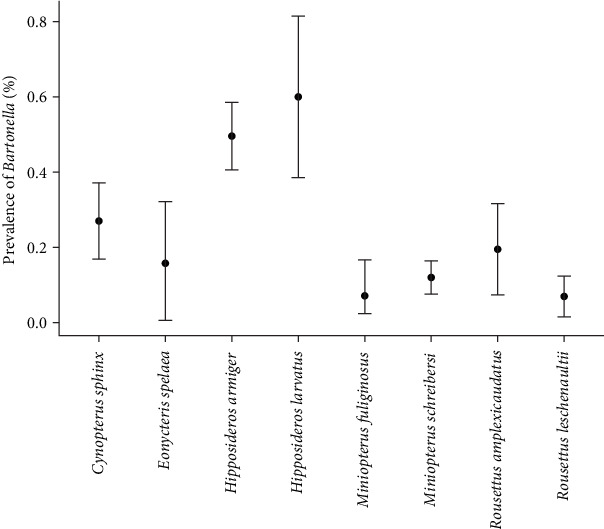
Prevalence of *Bartonella* among different bat species. Dots represent the prevalence rates of *Bartonella* for each bat species, while bar graphs indicate the 95% confidence intervals (95% CIs). Bat species with sample sizes fewer than 10 were excluded from the analysis.

Insectivorous bats had a significantly higher prevalence of *Bartonella* (25.99%, 98/377) compared to fruit-eating bats (17.86%, 40/224; *χ*^2^ = 5.260, *p* = 0.022). Regional prevalence rates were Yingjiang (52.98%, 80/151), Ruili (14.69%, 31/211), and Gengma (11.30%, 27/239). Prevalence in Yingjiang was significantly higher than in both Ruili (*χ*^2^ = 60.688, *p* < 0.001) and Gengma (*χ*^2^ = 80.759, *p* < 0.001), while the difference between Ruili and Gengma was not statistically significant (*χ*^2^ = 1.150, *p* = 0.283; Table 2, Figure 2).

Multifactorial logistic regression analysis identified bat species as the main factor influencing *Bartonella* prevalence. The odds of *Bartonella* infection in *H. armiger* were 5.285 times that in *E. spelaea* (*p* = 0.011) and 6.687 times higher in *H. larvatus* than in *E. spelaea* (*p* = 0.025; Supporting Information 2: Table S2 and Supporting Information 3: Figure S1).

### 3.2. Characteristics of *Bartonella* in Bat Ectoparasites

A total of 31 bat flies and one bat tick were collected in Yingjiang and Gengma Counties, belonging to two orders, three families, and four genera. These ectoparasites were grouped into eight pools for molecular analysis (Table 1). After nPCR for the *gltA* gene, a total of five reaction pools were detected with positive amplification of *Bartonella* (*rpoB* gene was not analyzed here because of unsuccessful amplification), with an overall prevalence of 62.50% (5/8, 95% CI: 24.50%−91.50%). Four out of six reaction pools of *Nycteribiidae* tested positive for *Bartonella* DNA amplification, with a prevalence of 50% (4/8). And then it also detected in one *Streblidae* reaction pool, but not in ticks.

### 3.3. Detection of *Bartonella* sp. in Bats and Ectoparasites

#### 3.3.1. Sequence Comparison and Phylogenetic Analysis Based on the *gltA* Gene of *Bartonella* sp. in Bats, Ectoparasites, and Strains Isolated From Bats

**Figure 3:**
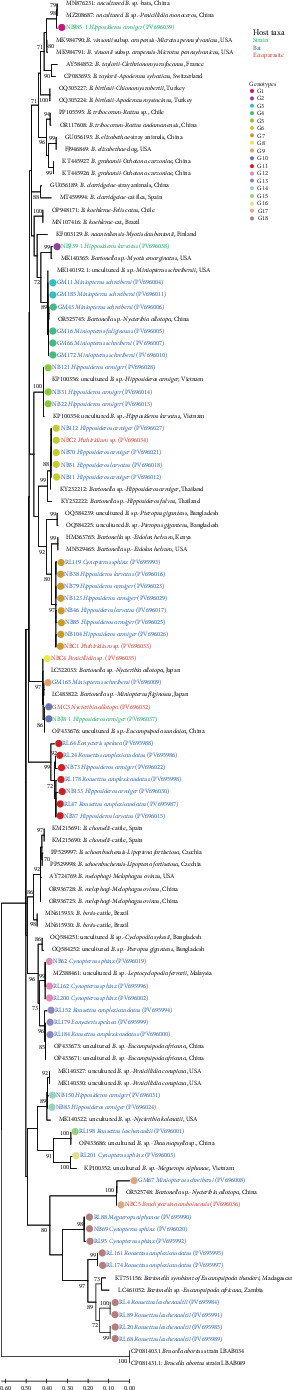
Phylogenetic classification of *Bartonella* species based on bat *gltA* gene sequences. The phylogenetic tree was constructed using the maximum likelihood method under the T92 + G model (Tamura 3-parameter + gamma distribution). Bootstrap values were calculated with 1000 iterations, and values greater than 70% are displayed on the tree.

We compared the 56 valid sequences obtained with the nucleotide sequences of *Bartonella* in GenBank and found that the identity ranged from 74.21% to 100.00% (Supporting Information 4: Table S3), revealing at least 18 genotypes (Figure 3). It is worth noting that from the analysis of 56 sequences obtained from all samples, we found that some genotypes had a high level of similarity: G1 is a strain isolated from a blood sample of *H. armiger*, and its sequence is almost identical to that of uncultured *Bartonella* sp. from bats in Hubei Province, China (99.37% identity, e.g., GenBank accession numbers MK876231). G2 is a strain isolated from *H. larvatus*, which is almost identical to *Bartonella* sp. detected in *M. emarginatus* in the USA (99.37% identity, e.g., GenBank accession numbers MK140365). There are two G3 sequences from *M. schreibersi* were identical to uncultured *Bartonella* sp. from *M. schreibersi* in the USA (100.00% identity, e.g., GenBank accession numbers MK140192). The three *M. schreibersi* sequences and one *M. fuliginosus* sequence in G4 are 99.69%–100.00% identical to *Bartonella* sp. from *N. allopota* collected from bats in China (e.g., GenBank accession numbers OR525745). There are three sequences from *H. armiger* in G5 were also completely identical to uncultured *Bartonella* sp. from *H. armiger* in Vietnam (100.00% identity, e.g., GenBank accession numbers KP100356). G6 includes four sequences from bats (three from *H. armiger* and one from *H. larvatus*) and one sequence from an ectoparasite of bats (*Phthiridium sp*. pool), which had 99.69% identity with *Bartonella* sp. from *H. armiger* in Thailand (e.g., GenBank accession numbers KY232212).

In addition, the G7 dataset comprises eight sequences: seven from bats (four from *H. armiger*, one from *H. larvatus*, and one from *C. sphinx*), and one from bat ectoparasites (*Phthiridium sp*. pool), which contains a *Bartonella* sp. strain isolated from *E. helvum* in Kenya that is 94.65%–95.28% identical to the reference strain (GenBank accession number HM363765). There are two sequences, G8 and G9, one from *Penicillidia* sp. pool and one from *M. schreibersi*, which clustered together with *Bartonella* sp. from *M. fuliginosus* and its ectoparasite *N. allopota* from Japan (100.00% identity, e.g., GenBank accession numbers LC522033 and LC483822). G10 includes one sequence from the *N. allotopa* pool collected from bats and one sequence from a strain isolated from *H. armiger*, both of which were identical to uncultured *Bartonella* sp. from ectoparasites (*E. sundaica*) of bats in Yunnan, China (100.00% identity, e.g., GenBank accession numbers OP433676).

Furthermore, G12 and G13 contain six sequences (three from *C. sphinx*, two from *R. amplexicaudatus*, and one from *E. spelaea*) that cluster with uncultured *Bartonella* sp. detected in ectoparasites of bats from Malaysia and China (98.12%–100.00% identity, e.g., GenBank accession numbers MZ388461 and OP433673). G14 consists of 2twosequences from *H. armiger* that are almost identical to uncultured *Bartonella* sp. from the ectoparasite *P. conspicua* of bats from the USA (99.06% identity, e.g., GenBank accession numbers MK140327). The two sequences of G15 and G16 (hosts are *R. amplexicaudatus* and *C. sphinx*) were grouped with uncultured *Bartonella* sp. of *Thaumapsylla* sp., an ectoparasite of Chinese bats, and *M. niphanae* in Vietnam, with identities of 100.00% and 96.86%, respectively (e.g., GenBank accession numbers OP433686 and KP100352). G17 has one sequence from *M. schreibersi* and one sequence from a bat ectoparasite (*B. amboinensis* pool), which also had high identity with *Bartonella* sp. on *N. allopota* collected from bats in Yunnan, China (identity of 94.65%–99.69%, e.g., GenBank accession numbers OR525748). The nine sequences of G18 are relatively new, and the hosts are mainly fruit bats (including *M. niphanae*, *C. sphinx*, *R. amplexicaudatus*, and *R. leschenaultii*), which have the highest identity with *Bartonella symbiont of Eucampsipoda theodori* from Madagascar, at around 90%.

The remaining new sequences are classified as G11, which has a low level of identity with *Bartonella* in GenBank and the highest identity with uncultured *Bartonella* sp. from *S. caffer* found in Africa (87.42%–89.62% identity, e.g., GenBank accession numbers MF774324). According to phylogenetic analysis of the *gltA* gene, it forms a new branch in the phylogenetic tree. Furthermore, Becker et al.'s [41] research on *Bartonella* in vampire bats shows that the relatedness between *Bartonella* in bats and ruminants is low, despite being classified as a branch. Therefore, based on the conventional 95% sequence identity threshold for bacterial species classification according to Goris et al. [42] and the critical value of less than 96.0% nucleotide identity in the *gltA* gene according to Scola et al. [27], we believe that a new species of *Bartonella* prevalent in bats has been discovered in this study, which we named “*Candidatus Bartonella dianxisis*.”

#### 3.3.2. Sequence Comparison and Phylogenetic Analysis Based on the *rpoB* Gene of Bartonella sp. in Bats, Ectoparasites, and Strains Isolated From Bats

**Figure 4:**
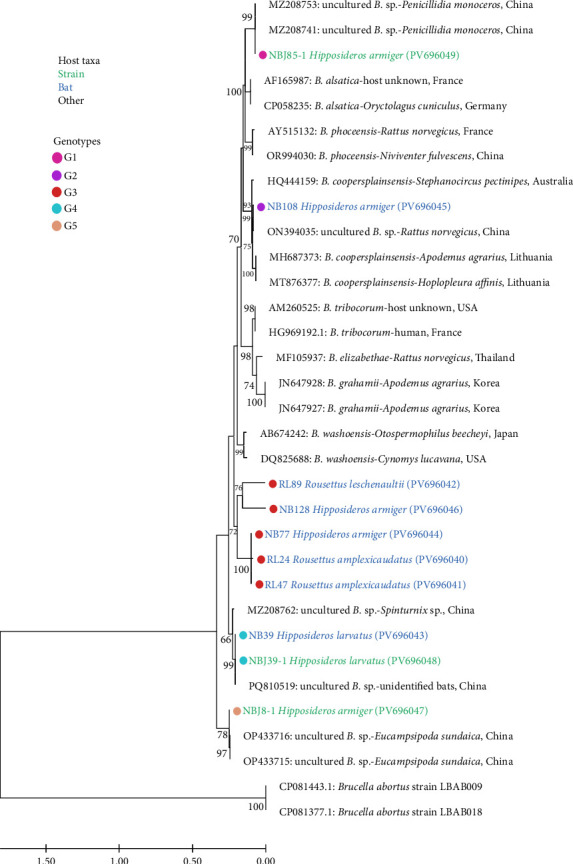
Phylogenetic classification of *Bartonella* species based on bat ectoparasites *rpoB* gene sequences. The phylogenetic tree was constructed using the maximum likelihood method under the T92 + G model (Tamura 3-parameter + gamma distribution). Bootstrap values were calculated with 1000 iterations, and values greater than 70% are displayed on the tree.

This study obtained a total of 10 *rpoB* gene sequences of *Bartonella*. Nucleotide sequence alignment of *Bartonella* was performed in GenBank, and the identity ranged from 79.58% to 100.00% (Supporting Information 5: Table S4). There were a total of five genotypes (Figure 4). Through sequence alignment and phylogenetic analysis, we found that four genotypes had a high level of identity with *Bartonella* in GenBank: G1 is a sequence isolated from *H. armiger*, which is almost identical to uncultured *Bartonella* sp. from *P. monoceros* collected from bats in Hubei Province, China (99.43% identity, e.g., GenBank accession numbers MZ208753). G2 has one sequence from *H. armiger* with 99.62% identity to uncultured *Bartonella* sp. detected in *R. norvegicus* in Guangzhou, China (e.g., GenBank accession numbers MZ208753). G4 has two sequences, one from *H. larvatus* and one from a strain isolated from this host, which clustered with uncultured *Bartonella* sp. from unidentified bats in Yunnan, China, with an identity of 99.81%–100.00% (e.g., GenBank accession numbers PQ810519). There is one sequence from G5, which came from *H. armiger*, showed a high level of identity with uncultured *Bartonella* sp. from *E. sundaica* collected from bats in Yunnan Province, China (99.43% identity, e.g., GenBank accession numbers OP433716).

The remaining five sequences are grouped in G3, whose hosts mainly include *H. armiger*, *R. amplexicaudatus*, and *R. leschenaultii*. The identity with *Bartonella* in GenBank is relatively low, but the highest identity was found with *Bartonella* sp. from *E. helvum* in Ghana, Africa (83.78%–90.46% identity, e.g., GenBank accession numbers KM215201). Based on *rpoB* gene phylogenetic analysis, it formed a separate branch in the phylogenetic tree, which is consistent with the results of the previous *gltA* gene study. Therefore, based on the conventional 95% sequence identity threshold in bacterial species classification and the study by Scola et al. [27], which is below the 95.4% threshold for *rpoB* gene nucleotide identity, the findings of this study on “*Candidatus Bartonella dianxisis*” are confirmed.

### 3.4. Tissue Tropism of *Bartonella* sp. in Bats

A qPCR standard curve was established using a series of plasmid dilutions ranging from 1.00 × 10^4^ to 1.00 × 10^8^ copies/µL. The Ct values for these plasmid dilutions were plotted, demonstrating a stable linear relationship between plasmid concentrations and their corresponding average Ct values (Supporting Information 6: Figure S2 and Supporting Information 7: Table S5). The minimum detectable copy number for the *Bartonella*-positive standard plasmid was 1.00 × 10^2^ copies/µL, highlighting the superior sensitivity of the qPCR method—100-fold higher than cPCR (Supporting Information 8: Figure S3 and Supporting Information 9: Figure S4).

**Figure 5:**
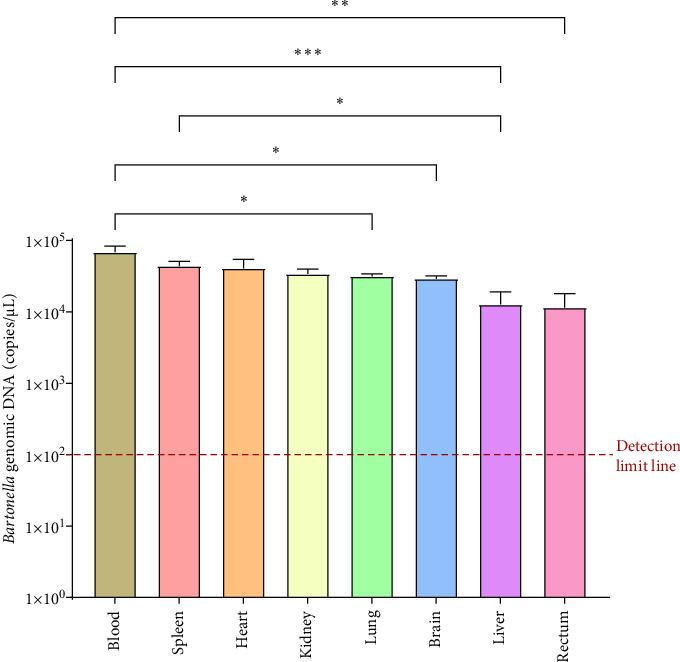
Tissue tropism of *Bartonella* in positive samples. The *Bartonella* load was measured across various tissues, including blood, spleen, heart, brain, kidney, lung, liver, and intestine, from 18 positive samples. Data are presented as mean ± standard error in copes/μL. Significance testing were conducted using one-way ANOVA (*⁣*^*∗*^*p* < 0.05, *⁣*^*∗∗*^*p* < 0.01, *⁣*^*∗∗∗*^*p* < 0.001). The red dotted line indicates the detection limits of qPCR established in this study.

A total of 18 bat samples, each with adequate blood and tissue samples, were analyzed for *Bartonella* copy numbers using qPCR. The mean copy numbers (ranked highest to lowest) were as follows: 6.87 × 10^4^ copies/μL (95% CI: 4.56 × 10^4^–9.70 × 10^4^) in blood, 5.26 × 10^4^ copies/μL (95% CI: 2.10 × 10^4^–6.89 × 10^4^) in the spleen, 4.08 × 10^4^ copies/μL (95% CI: 2.03 × 10^4^–3.66 × 10^4^) in heart, 3.49 × 10^4^ copies/μL (95% CI: 1.54 × 10^4^–4.53 × 10^4^) in the kidney, 3.41 × 10^4^ copies/μL (95% CI: 2.40 × 10^4^–4.10 × 10^4^) in the lung, 3.15 × 10^4^ copies/μL (95% CI: 1.66 × 10^4^–6.25 × 10^4^) in the brain, 1.27 × 10^4^ copies/μL (95% CI: 0.26 × 10^4^–3.97 × 10^4^) in liver, and 1.15 × 10^4^ copies/μL (95% CI: 1.11 × 10^4^–2.30 × 10^4^) in rectum (Supporting Information 10: Table S6). One-way ANOVA revealed that *Bartonella* copy numbers were significantly higher in blood, spleen, kidney, and heart tissues compared to other tissues. Lung and brain tissues had intermediate copy numbers, while the liver and rectum had the lowest (Figure 5).

### 3.5. Isolation and Characterization of *Bartonella* sp.

A total of 42 *Bartonella*-positive bat blood samples (Ct < 38) from Yingjiang County were selected for culture. After inoculating these samples on TSA medium containing 5% sheep blood, colony growth was monitored daily. Colonies began to appear as early as 3 days and up to 30 days postinoculation. Initially, colonies were small, raised, off-white to transparent, with smooth or rough edges and shiny or waxy surfaces. Over time, amphotericin B resistance decreased, and stray bacterial colonies emerged. Subsequent purification produced larger colonies with varying morphologies-some cauliflower-like, others diffuse, nonadherent, or slightly adherent to the medium. When colonies were scraped, a rounded depression was observed, indicative of *Bartonella* growth into the medium (Supporting Information 11: Figure S5A). From these cultures, three strains were isolated, including two from *H. armiger* and one from *H. larvatus*. Using Giemsa staining, it observed under a light microscope using oil immersion at a magnification of 1000x, the isolated *Bartonella* appeared as tiny bluish-purple, rod-shaped, or bulbous structures. The organisms measured 1–3 μm in length and were observed as single entities, in pairs, or chains forming V- or Y-shapes (Supporting Information 11: Figure S5B).

## 4. Discussion

Bats (order Chiroptera) are natural hosts for a wide range of bacteria and viruses globally, such as Rhabdoviridae, Paramyxoviridae, Coronaviridae, *Bartonella* sp., *Leptospira* sp., Enterobacteriaceae and so on [43]. Their capacity to migrate across borders and even continents contributes significantly to the spread of zoonotic diseases. Previous studies have demonstrated that *Bartonella* species carried by bats can infect humans and cause disease [16, 20]. The diversity of arthropod species associated with bats further suggests that *Bartonella* may be transmitted through blood-sucking ectoparasites acting as vectors [16, 44, 45]. Understanding the interactions among *Bartonella*, bats, and their vectors is,therefore, crucial.

The overall detection rate of *Bartonella* in this study was 22.96%, which was lower than the reported rates of 64.00% in Bangladesh [46], 26.00% in Japan [47], 25.50% in Thailand [48], and 24.10% in Peru [49], but higher than that the 16.70% reported in Zambia [50], 10.70% in Costa Rica [51], and 3.39% in South Africa and Swaziland [52]. Within China, the prevalence observed was lower than in central (32.70%) [53], northern (25.20%) [54], and southwestern regions (56.40%) [55] but higher than in eastern China (11.10%) [56]. Such differences may be attributed to variations in geographic location, bat species diversity, and environmental conditions [45].

In this study, the detection rate varied among bat species. While *M. schreibersi*, *R. leschenaultii*, and *R. blythiwere* commonly sampled, their *Bartonella* detection rates were 12.02%, 6.98%, and 0%, respectively. Notably, higher detection rates were observed in *M. niphanae* (66.67%), *H. larvatus* (60.00%), and *H. armiger* (49.58%). These findings are consistent with a study from Vietnam, where *M. niphanae* had a detection rate of 50.00%, *H. larvatus* 60.00%, and *H. armiger* 33.33% [57]. However, discrepancies exist when comparing the detection rates of *M. schreibersi* in Taiwan (42.90%) [56], Italy (25.00%), and Hungary (33.30%) [58], likely due to regional differences. Furthermore, while a study from Yunnan Province reported a prevalence of 56.90% for *R. leschenaultii* [55], our findings of 6.98% for this species highlight the influence of sample sizes and geographic variability. This study represents the first investigation into *Bartonella* prevalence in bats along the China–Myanmar border, offering valuable insights into species-specific infection rates and emphasizing the need for expanded sampling and geographic coverage in future studies.

Bat species is shown as the primary factor influencing *Bartonella* prevalence. In this study, prevalence rates were highest in *M. niphanae* (66.67%), *H. larvatus* (60.00%), and *H. armiger* (49.58%). Logistic regression analyses revealed no significant correlations between *Bartonella* prevalence and factors such as bat weight or sex. These findings reinforce the species-specific nature of *Bartonella* infections. Although global studies have documented *Bartonella* in bats and their ectoparasites [19, 52, 59], limited research has focused on southwest China. In this study, *Bartonella* was detected in 62.50% of bat flies including *Nycteribiidae* and *Streblidae*. This rate exceeds the 19.80% detection rate reported in Brazilian bat ectoparasites [60] and the 54.05% prevalence in Southwest China [35] but is lower than the 66.70% reported in Malaysia [61]. Differences are probably due to the small sample sizes, regional factors, and species diversity. Kuang et al. [35] detected *Bartonella* in mites, fleas, ticks, and wingless bat flies, highlighting ectoparasites' role in spreading *Bartonella*. Similarly, our study identified *Bartonella* in bat flies. The high infection rates observed in insectivorous bats suggest a correlation between host-vector interactions and feeding behaviors [62]. According to phylogenetic analysis, it revealed the sequences of ectoparasites were grouped together with the sequences obtained from bats, supporting the concept of a “host-vector-pathogen” transmission cycle [63, 64].

The *ssrA* gene is one of the most commonly used target genes for rapid detection of *Bartonella*. In contrast, genes encoding RNA polymerase subunits (*rpoB*) and citrate synthase (*gltA*) provide strong evidence for *Bartonella* typing [27]. Phylogenetic analysis revealed that *Bartonella* in bats and their ectoparasites in this study had a diverse genotype, with specific *Bartonella* genotypes associated with bat species and ectoparasite species, though some *Bartonella* genotypes could infect multiple hosts. Previous studies in Yunnan Province, China, have shown that the *Bartonella* found in bats differ significantly from those found in cats, dogs, primates, and rodents. This suggests that the *Bartonella* found in bats form an independent group [55]. Research by McKee et al. [65] also supports the view that adaptation to bat hosts is the primary factor in the evolution of *Bartonella* species associated with bats. This suggests that the *Bartonella* genotypes obtained are commonly transmitted and evolve among bats and their ectoparasites in Asian and Southeast Asian countries, but does not support frequent transmission between bats and other hosts. This is consistent with the findings of Becker et al. [41] in North America.

Our research into two genes revealed that some branches of the evolutionary tree do not cluster with known *Bartonella* species. This finding is consistent with a previous study of bat ectoparasites [35], indicating the potential presence of new, unknown *Bartonella* species in bats and their ectoparasites. For instance, Lilley et al. [14] identified “*Candidatus Bartonella hemsuletiensis*” from *M. daubentonii* in Finland. Furthermore, Lin et al. [20] isolated '*Candidatus* Bartonella mayotimonensis' from patient samples with endocarditis. Lilley et al. [66] conducted a survey of this *Bartonella* species in bats in North America, confirming that it may originate from bats. Since the *gltA* and *rpoB* genes both have good discriminatory power [29, 67], the limited first-generation sequencing results enabled the preliminary identification of *Bartonella*. Based on the conventional 95% sequence identity threshold for bacterial species classification by Goris et al. [42] and the critical values proposed by Scola et al. [27] of less than 96.0% nucleotide identity for the *gltA* gene and less than 95.4% nucleotide identity for the *rpoB* gene, we believe that a new species of *Bartonella* prevalent in bats has been discovered in this study. As the study area is located in western Yunnan Province, China, on the border with Myanmar, the new species has been named “*Candidatus Bartonella dianxisis*.” However, further in-depth studies on “*Candidatus Bartonella dianxisis*” are needed in terms of multigene locus sequencing, genetic evolutionary genomics, and pathogenic biology.

Our findings revealed a specific tissue tropism of *Bartonella*, with higher bacterial loads observed in the blood and spleen compared to other tissues (*p* < 0.05). This aligns with previous studies in small mammals and underscores the importance of these tissues in *Bartonella* biology.

Using an optimized culture method based on Liu et al. [38], we isolated three *Bartonella* strains. The isolation rate of 6.25%, which is lower than those reported in Zambian bats (16.70%) [50] and Nigerian bats (15.50%) [68]. This may be due to differences in geographical environments, species, and isolation methods between different studies, leading to varying results. Future studies on the strains isolated in this study should expand the number of housekeeping genes, such as 16S – 23S rRNA, *ribC*, ITS, and *ftsZ*, and obtain the whole genome through high-throughput sequencing to strengthen strain characterization.

## 5. Conclusion

In summary, we studied the prevalence, genetic diversity, and tissue tropism of *Bartonella* in bats and their ectoparasites in the China–Myanmar border region, as well as its isolation. The prevalence of *Bartonella* was mainly determined by bat species, and a high prevalence rate was also observed in their ectoparasites, suggesting that research on the host-vector-pathogen relationship should be strengthened. *Bartonella* in bats and their ectoparasites exhibited high genetic diversity. Our analysis revealed that a new *Bartonella* infected in bats may be occurring, which we named “*Candidatus Bartonella dianxisis*.” Additionally, we isolated three *Bartonella* strains and, through quantitative studies, observed that *Bartonella* exhibits broad tissue tropism. The pathogenicity of most bat-transmitted *Bartonella* strains to humans remains unclear to date. These findings highlight the need for further research to elucidate the zoonotic potential and interspecies transmission risks of bat-transmitted *Bartonella*.

## Figures and Tables

**Table 1:** Distribution and detection of *Bartonella* in bat ectoparasites from the China–Myanmar border region.

Extracorporeal parasite species			Location	Number of ectoparasites	Pools (*N*)	Positivity	
Family	Genus	Species				*n*/*N*	%
Nycteribiidae	*Penicillidia*	*Penicillidia* sp.	Gengma county	5	1	0/8	0.00
		*Penicillidia* sp.		5	1	0/8	0.00
	*Nycteribia*	*Nycteribia allotopa*		4	1	1/8	12.50
	*Phthiridium*	*Phthiridium* sp.	Yingjiang county	5	1	1/8	12.50
		*Phthiridium* sp.		5	1	1/8	12.50
	*Penicillidia*	*Penicillidia* sp.		4	1	1/8	12.50
Streblidae	*Brachytarsina*	*Brachytarsina amboinensis*		3	1	1/8	12.50
Ixodidae	*Ixodes*	*Ixodes collaris*		1	1	0/8	0.00
Total				32	8	5/8	62.50

**Table 2:** Distribution and detection of *Bartonella* in bats from the China–Myanmar border region (qPCR for *ssrA* gene and nPCR for *gltA* gene).

Bat species	Composition ratio (%)	Locations							
		Yingjiang county		Ruli county		Gengma county		Total	
		qPCR (%)	nPCR (%)	qPCR (%)	nPCR (%)	qPCR (%)	nPCR (%)	qPCR (%)	nPCR (%)
*Miniopterus schreibersi*	34.61 (208/601)	—	—	—	—	12.02 (25/208)	3.37 (7/208)	12.02 (25/208)	3.37 (7/208)
*Hipposideros armiger*	19.80 (119/601)	50.00 (59/118)	11.86 (14/118)	—	—	0.00 (0/1)	0.00 (0/1)	49.58 (59/119)	11.76 (14/119)
Rousettus leschenaultii	14.31 (86/601)	—	—	6.98 (6/86)	5.81 (5/86)	—	—	6.98 (6/86)	5.81 (5/86)
*Cynopterus sphinx*	12.31 (74/601)	66.67 (8/12)	16.67 (2/12)	24.19 (12/62)	8.06 (5/62)	—	—	27.03 (20/74)	9.46 (7/74)
*Rousettus amplexicaudatus*	6.82 (41/601)	—	—	19.51 (8/41)	17.07 (7/41)	—	—	19.51 (8/41)	17.07 (7/41)
Miniopterus fuliginosus	4.66 (28/601)	—	—	—	—	7.14 (2/28)	3.57 (1/28)	7.14 (2/28)	3.57 (1/28)
*Hipposideros larvatus*	3.33 (20/601)	60.00 (12/20)	20.00 (4/20)	—	—	—	—	60.00 (12/20)	20.00 (4/20)
*Eonycteris spelaea*	3.16 (19/601)	—	—	15.79 (3/19)	10.53 (2/19)	—	—	15.79 (3/19)	10.53 (2/19)
*Megaerops niphanae*	0.50 (3/601)	—	—	66.67 (2/3)	33.33 (1/3)	—	—	66.67 (2/3)	33.33 (1/3)
Rhinolophus blythi	0.33 (2/601)	—	—	—	—	0.00 (0/2)	0.00(0/2)	0.00(0/2)	0.00 (0/2)
*Macroglossus sobrinus*	0.17 (1/601)	100.00 (1/1)	0.00 (0/1)	—	—	—	—	100.00 (1/1)	0.00 (0/1)
Total	100.00 (601/601)	52.98 (80/151)	13.25 (20/151)	14.69 (31/211)	9.48 (20/211)	11.30 (27/239)	3.35 (8/239)	22.96 (138/601)	7.99 (48/601)

## Data Availability

The nucleotide sequences from *Bartonella* species, hosts, and ectoparasites described in this study have been deposited into the NCBI GenBank database (http://blast.ncbi.nlm.nih.gov/Blast.cgi, assessed on October 24, 2024) under accession numbers: PQ460893–PQ460958, PV667499–PV667524, PQ461921–PQ461925, PV658813, PV695984–PV696039, and PV696040–PV696049. The data are provided in the manuscript and supporting documents to this article and can be obtained from the corresponding author upon reasonable request.

## Supporting Information

Supporting Information 1 Table S1. Oligonucleotides, cycling conditions, and reaction systems used in quantitative real-time PCR and nested PCR assays based on *ssrA*, *gltA*, and *rpoB* genes for *Bartonella* molecular characterization

Supporting Information 2 Table S2. Logistic regression results for the effect of bat specie.

Supporting Information 3 Figure S1. Logistic regression results for the effect of bat species.

Supporting Information 4 Table S3. Comparisons of nucleotide identity and amino acid identity of *Bartonella gltA* gene (357 bp) from bat in this study.

Supporting Information 5 Table S4. Comparisons of nucleotide identity and amino acid identity of *Bartonella rpoB* gene (603 bp) from bat in this study.

Supporting Information 6 Figure S2. Standard curve.

Supporting Information 7 Table S5. Based on three repetitive qPCR experiments, the Ct values, slope, and amplification efficiency for each experiment.

Supporting Information 8 Figure S3. Sensitivity test results.

Supporting Information 9 Figure S4: Results of conventional PCR against diluted standard plasmids.

Supporting Information 10 Table S6. *Bartonella* species copies in tissues of bats.

Supporting Information 11 Figure S5: Isolated culture of *Bartonella*.
