# Supplementary material for: Molecular Detection and Isolation of Bartonella Species in Bats and Their Ectoparasites Along the China–Myanmar Border
Source: Transbound Emerg Dis. 2025 Aug 25;2025:5517852. doi: 10.1155/tbed/5517852 (PMC12401608; doi:10.1155/tbed/5517852)
Supplement: Supporting Information 1 — Table S1. Oligonucleotides, cycling conditions, and reaction systems used in quantitative real-time PCR and nested PCR assays based on ssrA, gltA, and rpoB genes for Bartonella molecular characterization [file 5517852.f1.docx]

| Target gene | Oligonucleotides (5′−3′) | Cycling conditions | reaction system | Size (bp) |
| --- | --- | --- | --- | --- |
| *ssrA*  (small stable R-NA) | F: GCTATGGTAATAAATGGACAATGAAATAA | 95 °C for 30 s  45 cycles of 95 ℃ for 10 s, 53 ℃ for 1 min  Acquisition of fluorescence signals during annealing | reaction mixture (20 μL total) comprised 10 μL of 2 × AceQ Universal U + Probe Master Mix V2, 0.4 μL each of F and R, and Probe (10 μM), 7.8 μL RNase-Free ddH2O, and 1 μL of DNA template | 301bp |
|  | R: GCTTCTGTTGCCAGGTG |  |  |  |
|  | Probe: FAM-ACCCCGCTTAAACCTGCGACG-BHQ1 |  |  |  |
| *gltA*  (citrate synthase gene) | First reaction:  443F: GCTATGTCTGCATTCTATCA | 95 °C for 3 min  35 cycles of 95 ℃ for 15 s, 48 ℃ for 15 s and 72 ℃ for 30 s  72 ℃ for 5 min | reaction mixture (25 μL total) comprised 12.5 μL of 2 × Rapid Taq Master Mix, 1μL each of F and R (10 μM), 7.5 μL RNase-Free ddH2O, and 3 μL of DNA template | 800bp |
|  | 1210R: GATCYTCAATCATTTCTTTCCA |  |  |  |
|  | Second reaction:  781F: GGGGACCAGCTCATGGTGG | 94 °C for 3 min  35 cycles of 94 ℃ for 15 s, 54 ℃ for 15 s and 72 ℃ for 30 s  72 ℃ for 5 min | reaction mixture (25 μL total) comprised 12.5 μL of 2 × Rapid Taq Master Mix, 1μL each of F and R (10 μM), 9.5 μL RNase-Free ddH2O, and 1 μL of first reaction products template | 357bp |
|  | 1137R: AATGCAAAAAGAACAGTAAACA |  |  |  |
| *rpoB*  (polymerase beta‐subunit  gene) | First reaction:  1400F: CGCATTGGCTTACTTCGTAT | 95 °C for 3 min  35 cycles of 95 ℃ for 15 s, 48 ℃ for 15 s and 72 ℃ for 30 s  72 ℃ for 5 min | reaction mixture (25 μL total) comprised 12.5 μL of 2 × Rapid Taq Master Mix, 1μL each of F and R (10 μM), 7.5 μL RNase-Free ddH2O, and 3 μL of DNA template | 825bp |
|  | 2300R: GTAGACTGATTAGAACGCTGG |  |  |  |
|  | Second reaction:  1596F: CGCATTATGGTCGTATTTGTCC | 94 °C for 3 min  30 cycles of 94 ℃ for 15 s, 51 ℃ for 15 s and 72 ℃ for 30 s  72 ℃ for 5 min | reaction mixture (25 μL total) comprised 12.5 μL of 2 × Rapid Taq Master Mix, 1μL each of F and R (10 μM), 9.5 μL RNase-Free ddH2O, and 1 μL of first reaction products template | 603bp |
|  | 2300R: GTAGACTGATTAGAACGCTGG |  |  |  |

**Table S1** Oligonucleotides, cycling conditions and reaction systems used in quantitative real-time PCR and nested PCR assays based on *ssrA*, *gltA* and *rpoB* genes for *Bartonella* molecular characterization
