# Supplementary material for: Molecular Detection and Isolation of Bartonella Species in Bats and Their Ectoparasites Along the China–Myanmar Border
Source: Transbound Emerg Dis. 2025 Aug 25;2025:5517852. doi: 10.1155/tbed/5517852 (PMC12401608; doi:10.1155/tbed/5517852)
Supplement: Supporting Information 3 — Figure S1. Logistic regression results for the effect of bat species. [file 5517852.f3.docx]

**Figure S1** Logistic regression results for the effect of bat species (Visualization of OR 95%CI chart), Dots represent OR and bar graphs represent 95% confidence intervals (95% CI) (species with sample sizes less than ten were not included in the analysis)
