# Supplementary material for: Molecular Detection and Isolation of Bartonella Species in Bats and Their Ectoparasites Along the China–Myanmar Border
Source: Transbound Emerg Dis. 2025 Aug 25;2025:5517852. doi: 10.1155/tbed/5517852 (PMC12401608; doi:10.1155/tbed/5517852)
Supplement: Supporting Information 6 — Figure S2. Standard curve. [file 5517852.f6.docx]

**Figure S2 Standard curve**. The equation of the standard curve is y = -3.542x + 48.39, with a correlation coefficient (R2) of 0.9997. The slope is -3.542, and the amplification efficiency (E%) is 91.57%.
