# Supplementary material for: Molecular Detection and Isolation of Bartonella Species in Bats and Their Ectoparasites Along the China–Myanmar Border
Source: Transbound Emerg Dis. 2025 Aug 25;2025:5517852. doi: 10.1155/tbed/5517852 (PMC12401608; doi:10.1155/tbed/5517852)
Supplement: Supporting Information 7 — Table S5. Based on three repetitive qPCR experiments, the Ct values, slope, and amplification efficiency for each experiment. [file 5517852.f7.docx]

**Table S5:** Based on three repetitive qPCR experiments, the Ct values, slope, amplification efficiency for each experiment

| **Dilution** | **Ct1** | **Ct2** | **Ct3** | **Ct mean** |
| --- | --- | --- | --- | --- |
| 1×10^8^ | 23.378 | 19.561 | 20.013 | 20.984 |
| 1×10^7^ | 24.945 | 24.577 | 24.322 | 24.615 |
| 1×10^6^ | 28.197 | 27.851 | 28.543 | 28.197 |
| 1×10^5^ | 31.784 | 31.817 | 31.751 | 31.784 |
| 1×10^4^ | 35.108 | 35.112 | 35.107 | 35.109 |
|  |  |  |  |  |
| **Slope** | -3.030 | -3.834 | -3.762 | -3.542 |
| **R^2^** | 0.9845 | 0.9942 | 0.9952 | 0.9997 |
| **Amplification efficiency (%)** | 113.81 | 82.32 | 84.42 | 91.57 |
