# Supplementary figures and images for: Molecular Detection and Isolation of Bartonella Species in Bats and Their Ectoparasites Along the China–Myanmar Border
Source: Transbound Emerg Dis. 2025 Aug 25;2025:5517852. doi: 10.1155/tbed/5517852 (PMC12401608; doi:10.1155/tbed/5517852)

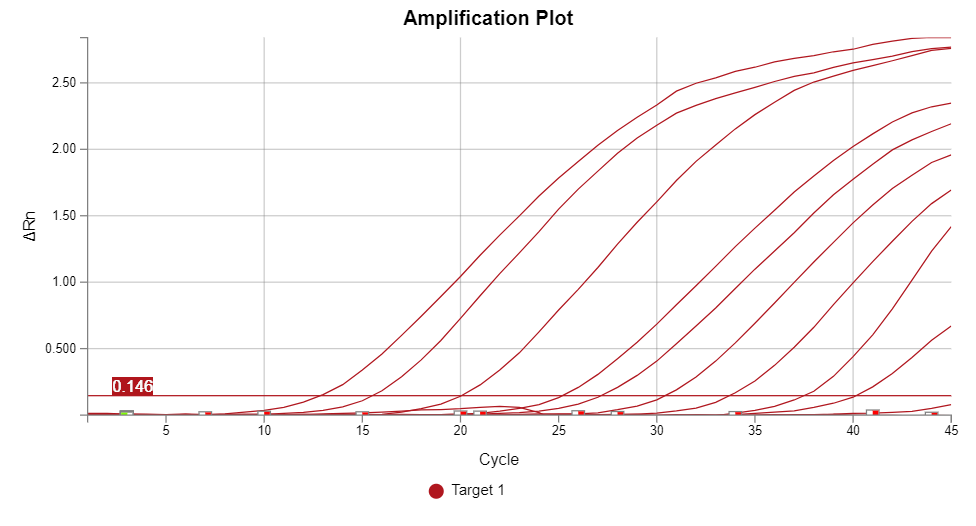


1

2

3

4

5

6

7

8

9

10

**Figure S3 Sensitivity test results**. Copies ranged from 1.0×1010 to 1.0×101copies/L

Supplement: Supporting Information 8 — Figure S3. Sensitivity test results. [file 5517852.f8.docx]
