# Supplementary material for: Molecular Detection and Isolation of Bartonella Species in Bats and Their Ectoparasites Along the China–Myanmar Border
Source: Transbound Emerg Dis. 2025 Aug 25;2025:5517852. doi: 10.1155/tbed/5517852 (PMC12401608; doi:10.1155/tbed/5517852)
Supplement: Supporting Information 9 — Figure S4: Results of conventional PCR against diluted standard plasmids. [file 5517852.f9.docx]

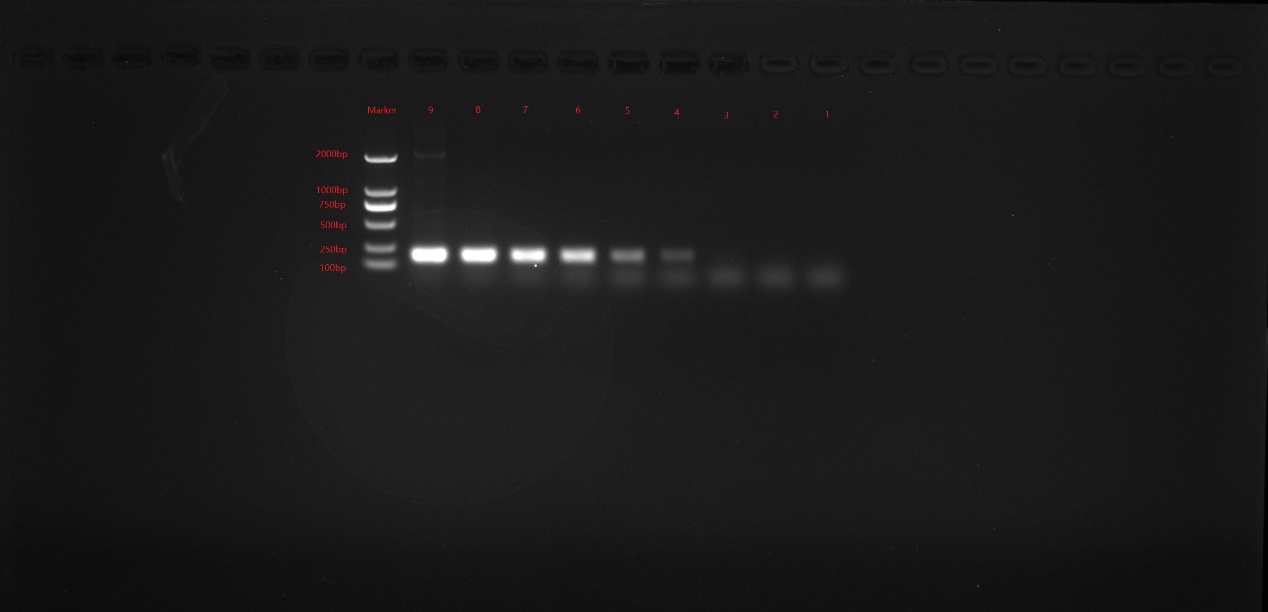


**Figure S4 Results of Conventional PCR against diluted standard plasmids**. M: Trans2K DNA Marker; 9 - 2: 1.0×10 ^9^ - 1.0×10 ^2^ ; 1: negative control (Using ddH2O as a template)
