# Supplementary material for: Molecular Detection and Isolation of Bartonella Species in Bats and Their Ectoparasites Along the China–Myanmar Border
Source: Transbound Emerg Dis. 2025 Aug 25;2025:5517852. doi: 10.1155/tbed/5517852 (PMC12401608; doi:10.1155/tbed/5517852)
Supplement: Supporting Information 10 — Table S6. Bartonella species copies in tissues of bats. [file 5517852.f10.docx]

**Table S6** *Bartonella* species copies in tissues of bats.

|  | Blood | Spleen | Heart | Kideny | Lung | Brain | Liver | Rectum |
| --- | --- | --- | --- | --- | --- | --- | --- | --- |
| Mean (copies/μL) | 6.87×10^4^ | 5.26×10^4^ | 4.08×10^4^ | 3.41×10^4^ | 3.15×10^4^ | 2.91×10^4^ | 1.27×10^4^ | 1.15×10^4^ |
| SEM (copies/μL) | 1.51×10^4^ | 7.52×10^3^ | 1.33×10^3^ | 2.90×10^3^ | 5.43×10^3^ | 2.60×10^3^ | 6.30×10^3^ | 6.53×10^3^ |
