# Supplementary material for: Molecular Detection and Isolation of Bartonella Species in Bats and Their Ectoparasites Along the China–Myanmar Border
Source: Transbound Emerg Dis. 2025 Aug 25;2025:5517852. doi: 10.1155/tbed/5517852 (PMC12401608; doi:10.1155/tbed/5517852)
Supplement: Supporting Information 11 — Figure S5: Isolated culture of Bartonella. [file 5517852.f11.docx]

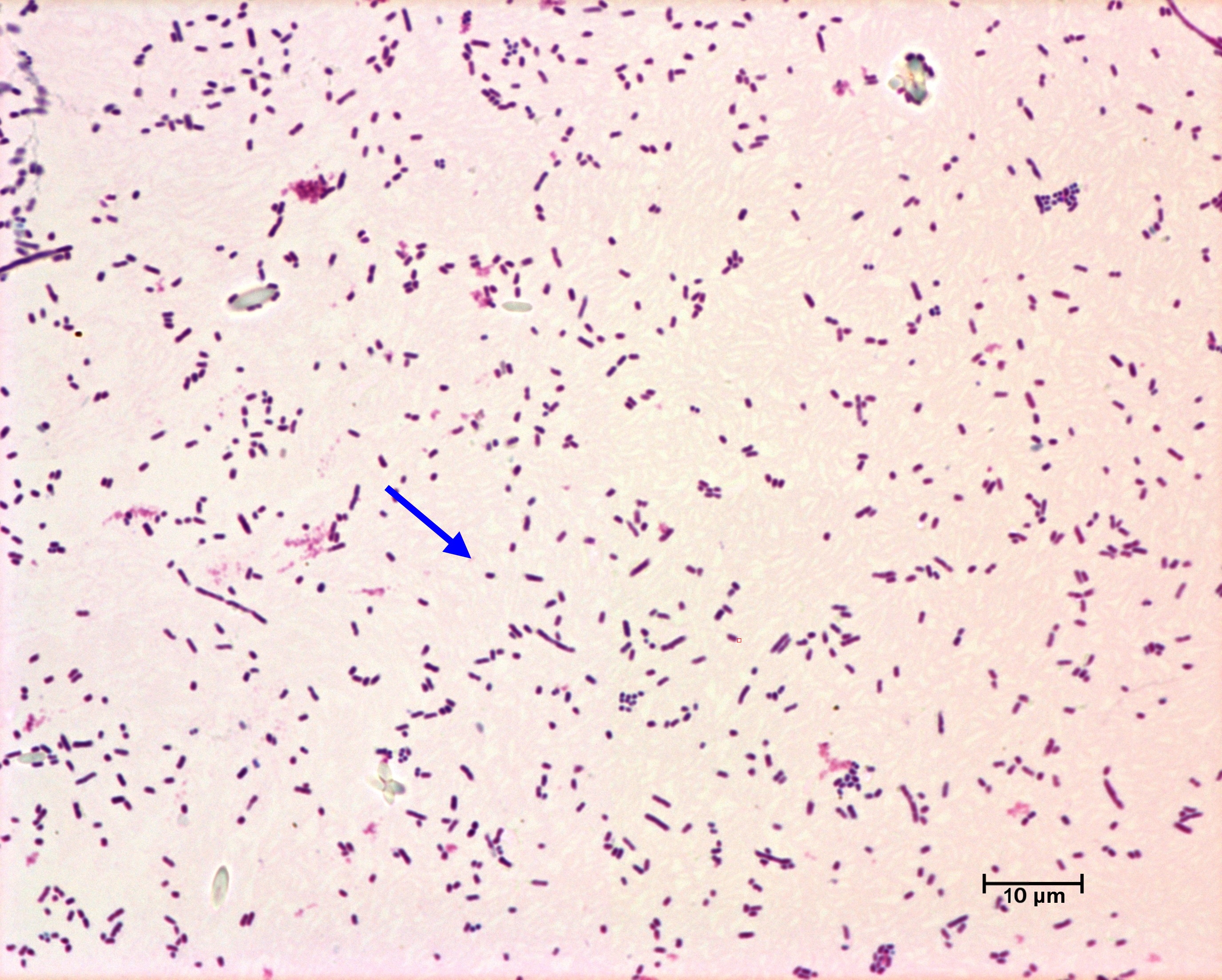

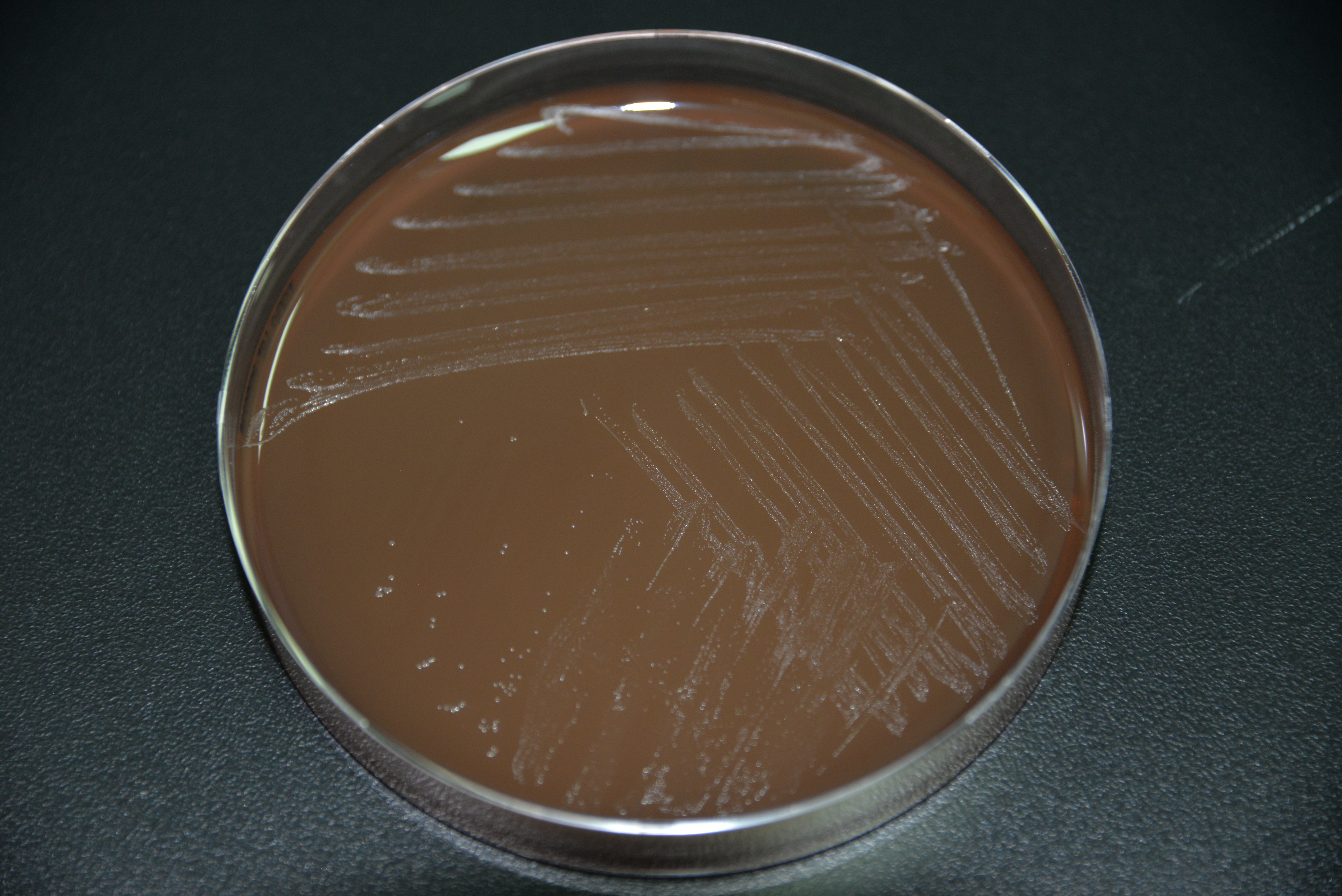


**A**

**B**

**Figure S5: Isolated culture of *Bartonella***.

(A) Purified *Bartonella* colonies 6 passages, grown on TSA medium containing 5% sheep blood.

(B) A single *Bartonella* bacterium, stained blue-purple with Giemsa (1000×).
